# Public Service Motivation and Turnover Intention: Testing the Mediating Effects of Job Attitudes

**DOI:** 10.3389/fpsyg.2020.01289

**Published:** 2020-06-23

**Authors:** Kai-Peng Gan, Yun Lin, Qiu Wang

**Affiliations:** School of Finance and Public Administration, Yunnan University of Finance and Economics, Kunming, China

**Keywords:** public service motivation, job attitudes, turnover intention, public sector, public employee

## Abstract

Research on the role of public service motivation (PSM) relating to work performance has been a significant topic in recent years; however, the relationship between PSM and job performance remains mixed. To investigate whether job attitudes mediate the effect of PSM on public employees’ turnover intention, this study integrated job satisfaction and organizational commitment into a single model. Based on a sample of 587 full-time Chinese public employees, our findings revealed that job satisfaction and organizational commitment, respectively, mediated the negative association between PSM and employees’ turnover intention. Multiple mediation analysis indicated that job satisfaction and organizational commitment sequentially mediated the effects of PSM on turnover intention. As a result, our findings suggested that public employees with high PSM levels preferred to stay in the public organizations. The theoretical and practical implications of our findings are discussed.

## Introduction

Perry and Wise (1990) argued that individuals with high levels of public service motivation preferred to seek a job within the public sector and to perform better in public sector work. Subsequently, a stream of empirical studies has focused on the relationship between PSM and job performance (Kim, 2012; Shim et al., 2017; Choi and Chun, 2018; Jin et al., 2018; Miao et al., 2019; Caillier, 2020). A substantial amount of literature has laid stress on the significance of PSM in boosting job performance, but “the role of intermediate variables, mediating the relationship between PSM and performance, is still unclear” (Perry et al., 2010). In PSM literature, there are two research perspectives on whether individual PSM is negatively related to their intentions to stay in the public sector. Some studies have explored the direct effect of PSM on employees’ turnover intention (Alonso and Lewis, 2001; Andersen and Søren, 2012; Choi and Chun, 2018), but more recent research has shed light on the link between PSM and turnover intention which was mediated by intermediate variables, including the person-organization fit (Bright, 2008; Kim, 2012; Gould-Williams et al., 2015), organizational commitment (Vandenabeele, 2009; Jin et al., 2018), organizational identification (Miao et al., 2019), social impact potential (van Loon et al., 2018) and meaningfulness of work (Zheng et al., 2020). However, the causality between PSM and turnover intention remains highly disputed. For example, findings by Bright (2008) confirmed a non-significant association between PSM and employees’ turnover intention, which was consistent with the results of Alonso and Lewis (2001). In contrast to Bright, some recent results have revealed that the relationship between PSM and employees’ job performance is significant when some mediators (e.g., P-O fit or organizational commitment) are taken into account (Kim, 2012; Gould-Williams et al., 2015; Jin et al., 2018).

In a word, most of the existing PSM literature does not provide strong evidence for the association between PSM and turnover intention. Furthermore, prior scholars have stressed the dominant role of P-O fit in the connection between PSM and employees’ work-related outcomes (Kim, 2012; Gould-Williams et al., 2015; Jin et al., 2018), but they have largely ignored the effects of other mediating variables, such as work attitudes and behavior (Vandenabeele, 2009). Mathieu et al. (2016) noted that despite the critical role of job satisfaction and organizational commitment in explaining turnover intention that has increasingly attracted attention in recent years, few studies have considered job satisfaction and organizational commitment together when presenting a structure turnover model. Therefore, it is essential to clarify the direct and indirect effects of PSM on employees’ turnover intention by incorporating mediators—job satisfaction and organizational commitment—into a single research model.

Our study aims to explore the effects of PSM on public employees’ turnover intention. The negative association between PSM and employees’ turnover intention has received little attention and demonstrated mixed findings (Bright, 2008; Kim, 2012; Jin et al., 2018). To investigate the inconsistent findings between PSM and employees’ turnover intention, researchers have laid stress on the cross-cultural comparison using diverse samples from different cultural context (Kim, 2017). Our study provides a new insight into the empirical research related to the links between PSM and turnover intention by shedding light on work-related behavior of Chinese public employees. As such, by focusing on Chinese public employees, our study extends the previous research that has been conducted with samples from Western countries. Another goal of the present study is to explore how public employees’ job attitudes mediate the effects of PSM on their turnover intention. To our knowledge, the current empirical findings revealed that the association between PSM and employees’ turnover intention was significantly mediated by employees’ P-O fit (Gould-Williams et al., 2015; Jin et al., 2018). However, researchers have rarely taken into account the role of other mediators (e.g., job satisfaction and organizational commitment) when discussing employees’ turnover intention in the public sector (Vandenabeele, 2009). Thus, we propose a turnover model that integrates job satisfaction, organizational commitment, as well as turnover intention. In doing so, our findings stress the importance of mediating role of public employees’ job attitudes in predicting the impact of PSM on turnover intention.

## Literature Review

### PSM and Turnover Intention

The concept of PSM that originated from Perry and Wise (1990) refers to “an individual’s predisposition to respond to motives grounded primarily or uniquely in public institutions or organization.” The work of Perry and Hondeghem (2008) shows that individuals with a high level of PSM will select jobs in the public sector for the sake of public benefits. Alternatively, Vandenabeele (2007) saw PSM as “belief, value, and attitudes that go beyond self-interest and organizational interest, that concern the interest of a larger political entity and that motivate individuals to act accordingly whenever appropriate.” Furthermore, PSM can be viewed “as a specific type of prosocial motivation” or intrinsic motivation for public employees (Georgellis and Tabvuma, 2010; Ritz et al., 2020). Importantly, the concept of PSM has been discussed and used outside the public sector, because PSM is found in commercial settings (O’Leary, 2019). Therefore, scholars proposed more generalized concepts of PSM. For example, Rainey and Steinbauer (1999) defined PSM as a “general, altruistic motivation to serve the interests of a community of people, a state, a nation or humankind.” While PSM is originally viewed as a prosocial motivation to do good for others and the society within public sector (Perry and Wise, 1990; Hondeghem and Perry, 2009), it has been an increasing recognition that PSM has been a growing concern in various areas and is further discussed outside the public sector (Bozeman and Su, 2015; O’Leary, 2019).

As such, PSM can be described as “autonomous types of motivation” (Houston, 2011) that will play a key role in affecting individual work-related behaviors. These views indicate that both internal motives and external context play a vital role in determining individual work-related behaviors, such as job performance, work effort, and organizational performance (Bright, 2007; van Loon, 2017). Previous empirical studies have found that work-related attitudes and behaviors of public employees are closely connected with PSM (Perry and Wise, 1990; Bright, 2007; Leisink and Steijn, 2008). Generally speaking, many believe that individuals tend to work in the public organizations and report high job satisfaction, job performance, and organizational commitment when they have high PSM levels (Perry, 1996; Moynihan and Pandey, 2007). With a large sample of employees in the Italian National Health Service, Belle (2013) argued that PSM significantly increased job performance among public employees. More recently, the work of Levitats and Vigoda-Gadot (2017) showed that PSM was positively linked with public employees’ job outcomes, such as job satisfaction, affective commitment, and service quality.

Generally speaking, turnover intention refers to “probability of employees leaving their organization” (Wynen et al., 2013). Although previous studies have suggested that employees’ turnover intention is largely determined by economic, individual, and organizational factors (Moynihan and Landuyt, 2008), PSM theory fundamentally assumes that those people with high PSM levels prefer to “seek membership in a public organization” to obtain opportunities to promote public interests (Perry and Wise, 1990; Vandenabeele, 2008). Some earlier studies have found that PSM has no direct and significant impact on turnover intention (Kim and Lee, 2007; Bright, 2008); however, most recent empirical findings have confirmed the negative relationship between PSM and turnover intention in the public sector (Leisink and Steijn, 2008; Shim et al., 2017; Choi and Chun, 2018). Based on a sample of 4,974 street-level bureaucrats, Shim et al. (2017) revealed that the direct effect of PSM on employees’ turnover intention was negative and significant. Similarly, Choi and Chun (2018) concluded that individuals who attach importance to social equity and altruism were not likely to quit a job within the public sector. Actually, the negative effect of red tape on employees’ turnover intention can be mitigated by PSM because public employees with high PSM levels have less intention to leave their jobs within the public sector (Quratulain and Khan, 2015). However, the empirical study of Liu et al. (2011) revealed that “only a self-sacrifice dimension of PSM was positively related to occupational intention” to seek membership in a public organization. Based on the previous discussions, we expect that PSM will play a vital role in employees’ turnover intention because public employees with high-PSM are more willing to contribute to the interests of others and society. Thus, it leads to the following hypothesis:

*Hypothesis 1:* PSM is negatively correlated with public employees’ turnover intention.

### The Mediating Role of Job Satisfaction

According to Locke (1976), job satisfaction can be seen as “a pleasure or positive emotional state resulting from the appraisal of one’s job or job experience.” An enormous amount of empirical research has explored the significance of job satisfaction and its consequences. According to the organizational equilibrium theory, employees intend to change jobs when they perceive that they have received less inducement from their organizations than their contributions to their job. Indeed, research has confirmed that employees’ job dissatisfaction increase their desirability to leave and, thereby, leads to a greater intent to leave (Huang et al., 2017; Zeffane and Melhem, 2017). With regard to its potential influence on organizations, job satisfaction generally has played a significant role in the decision of employees’ intention to quit, because it has been one of the “most critical predictors of turnover intention” (Choi and Chiu, 2017). At the organizational level, job satisfaction is closely related to the degree of absenteeism, job performance, turnover intention, and organizational citizenship behavior (Wright and Bonett, 2007). At the individual level, some empirical research revealed that job satisfaction had a positive relationship with individual life satisfaction, well-being, or mental health, but it also has a negative relationship with anxiety and depression (Christian et al., 2011; Zalewska, 2011). In the context of value pluralism, employees’ intention to leave the job not only is associated with work-relate factors, but it also is linked to personal cognition, such as career orientation, organizational identity, or job pressure. However, as an emotional state that resulted from job experience, job satisfaction plays an important role in work-related behaviors. To be specific, Khan and Aleem (2014) identified that job satisfaction was affected by salary, promotion opportunities, job security, or job demands and, thus, resulted in negative influences on an employee’s intention to leave the job. Obviously, employees will tend to change jobs when they are dissatisfied with the present jobs.

Although numerous work-related relevant factors can bring about job satisfaction, the relationship between PSM and job satisfaction has gained increased research attention in recent years (Wright and Pandey, 2008; Liu et al., 2015; Choi and Chun, 2018; Kjeldsen and Hansen, 2018). These results are based on the assumption that public organizations provide opportunities for those employees with high PSM levels to do good for others and society, which meets their altruistic needs and, subsequently, leads to higher job satisfaction (Perry et al., 2010; Andersen and Kjeldsen, 2013). Furthermore, the work of Kjeldsen and Andersen (2013) revealed that PSM is positively correlated with job satisfaction when employees perceive that “their jobs are useful to society and other people.” Consistent with Balfour and Wechsler’s earlier proposition, PSM has been identified as being directly and indirectly related to job satisfaction through the moderation of P-O fit (Liu et al., 2015; Kim, 2012). To be specific, based on empirical data collected in Chinese public sector, Liu et al. (2015) suggested that the impact of PSM on job satisfaction was significant if both P-O fit and needs-supplies fit were low. Similarly, using a sample of civil servants employed by local governments in Korea, Kim (2012) revealed that PSM was not only an important independent factor in job satisfaction, but it also had “an indirect effect on job satisfaction and organizational commitment through its influence on P-O fit.”

However, some earlier empirical research shows mixed findings regarding the association between PSM and job satisfaction. For example, Bright (2008) and Steijn (2008) argued that PSM has direct impact on work-related outcomes when P-O fit was considered. This was supported by Wright and Pandey (2008) who also claimed that PSM did not directly affect employees’ job satisfaction if P-O fit was integrated into the research model as a mediator. Nevertheless, most scholars argued that the effects of PSM on work-related outcomes could not be fully explained through P-O fit theory, because PSM theory had been widely validated within both public and private organizations (Christensen and Wright, 2011; Choi and Chun, 2018; Andersen and Kjeldsen, 2013). More recently, Taylor (2014) suggested that public employees “with high PSM levels tend to be more satisfied with their job than those with low PSM levels.” Further, Andersen and Kjeldsen (2013) findings also confirmed that the direct and positive effect of PSM on job satisfaction was significant in both public sector and private sector.

As one of the consequences of PSM in the public sector, job satisfaction may play a critical role in bringing about better performance, such as low intention to quit a job within the public sector, especially when individuals hold an altruistic need to serve others and the community. Although P-O fit is more commonly considered as an important mediator in the PSM-job performance relationship (Bright, 2008; Kim, 2012; Gould-Williams et al., 2015; Jin et al., 2018), job satisfaction may act as a possible mediating variable in the link. For instance, the findings by Vandenabeele (2009) revealed that PSM had an indirect impact on individual performance through normative and affective organizational commitment. In other words, an individual with high PSM levels is expected to experience a high level of job satisfaction, which, in turn, results in lower turnover intention. In sum, based on these arguments with regard to the significant relationships among PSM, job satisfaction and turnover intention, we propose that individuals with high levels of PSM will be satisfied with their job, and this increased job satisfaction will contribute to explain the relationship between PSM and employees’ turnover intention.

*Hypothesis 2*: Job satisfaction is negatively correlated with employees’ turnover intention.

*Hypothesis 3:* Job satisfaction mediates the negative link between employees’ PSM and their turnover intention.

### The Mediating Role of Organizational Commitment

Broadly speaking, organizational commitment is described as “a psychological state that characterizes the employee’s relationship with the organization” (Meyer et al., 1993). Allen and Meyer (1993) suggested that organizational commitment was an important predictor of turnover intention, because “it has implications for the decision to continue or discontinue membership in the organization.” A considerable number of studies have demonstrated that employees’ organizational commitment has a negative relationship with their intention to leave their organizations (Islam et al., 2015; Lambert et al., 2015; Yousaf et al., 2015; Gatling et al., 2016; Wombacher and Felfe, 2017). For instance, Wombacher and Felfe (2017) revealed that affective organizational commitment and team commitment reduced employees’ target-specific turnover intention and, therefore, committed employees were more likely to remain in their organizations, especially if their levels of team commitment were high. Actually, when employees’ desires and expectations are satisfied, they will shape more psychological attachment toward their organizations and are less likely to change jobs (Nazir et al., 2016). In line with this view, we believe that affective and normative commitment is negatively related to employees’ turnover intention in a Chinese organizational context. In the Chinese context, empirical studies showed that employees’ turnover intention was not only related to organizational commitment (Tian, 2014; Li et al., 2018) but negatively connected with the work environment (Wang, 2015), organizational culture (Ma et al., 2015), emotional exhaustion (Li et al., 2018), or psychological contract (Tang et al., 2017). As Li et al. (2018) pointed out, only affective commitment is a significant predictor of Chinese employees’ turnover intention. However, Tian (2014) argued that all components of organizational commitment were negatively related to employees’ turnover intention. Generally speaking, employees would like to maintain a durable attachment to their organizations and to show less intention to leave their organizations when they have higher organizational commitment.

Furthermore, as “a psychological state” to a particular organization (Allen and Meyer, 1993), organizational commitment has several significant antecedents, such as career identification, value congruence, PSM, and so on. The original basis of PSM theory suggests that individuals are motivated to do good for public service for particular reasons, among which the most important is “commitment to the public interest” (Perry, 1996). In general, PSM acts as the antecedent of organizational commitment, which indicates that PSM is positively associated with organizational commitment (Taylor, 2008; Vandenabeele, 2009; Kim, 2012; Caillier, 2016; Levitats and Vigoda-Gadot, 2017). According to Kim (2012), “as the levels of PSM in public employees increase, their loyalty and emotional identification with the organization that seeks public interests will also increase.” Potipiroon and Ford (2017) supported scholars’ arguments that ethical leadership and high-intrinsic motivation mediated the positive association between PSM and public employees’ organizational commitment. Similarly, Jin et al. (2018) suggested that the effect of PSM on organizational commitment was positive and significant, which in turn led to more positive attitudes and commitment toward the employees’ work.

Yet, other studies showed that the effect of PSM on organizational commitment depended on the intermediate variables to some extent (Kim et al., 2015). Kim et al. (2015), for example, found that “when employees’ affective commitment is high in a context where their values are consistent with their organization’s goals or missions, their level of PSM will increase.” That is to say, the levels of affective commitment among public employees will reinforce their pro-social behaviors and positive work value, which leads to more motivation to serve others and the public. In addition, grounded in P-O fit perspective, many scholars indicate that the relationship between PSM and work-related outcomes (e.g., organizational commitment) was partially mediated by P-O fit (Kim, 2012; Jin et al., 2018). As such, we can expect that public employees will exhibit more commitment to their organizations when they have high levels of PSM (Kim et al., 2013), because they can obtain opportunities in the public organization to contribute to the public (Luu, 2018).

Taken together, this stream of studies has suggested that PSM contributes to increase employees’ loyalty and affective identification to their organizations and, in turn, employees will prefer to stay in their organizations because a strong desire to remain in their job demonstrated commitment to the organization. As Vandenabeele (2009) described, both normative organizational commitment and affective organizational commitment mediated the association between PSM and individual work-related outcome. Similarly, Caillier (2015) also argued that organizational commitment mediated the association between PSM and government employees’ organizational citizenship behavior (e.g., whistle-blowing attitudes) in the United States. In line with these opinions, Jin et al. (2018) also argued that organizational commitment partially mediated the relationship between PSM and individual performance (e.g., organizational citizenship, institutional service) in public higher education. Although the negative effect of organizational commitment on turnover intention has been confirmed by extensive previous research, few studies have explore the association between PSM and employees’ turnover intention by examining the mediating role of organizational commitment. According to the previous research (Wright and Pandey, 2008; Vandenabeele, 2009; Jin et al., 2018), it is possible that PSM has indirect impact on work-related attitudes and behaviors through organizational commitment. As such, it is worthwhile to validate whether organizational commitment reinforces the ability of PSM to reduce employees’ turnover intention in the public sector. To test it, we propose the following hypotheses.

*Hypothesis 4*: Organizational commitment is negatively correlated with employees’ turnover intention.

*Hypothesis 5*: Organizational commitment mediates the negative link between employees’ PSM and their turnover intention.

### The Chain-Mediating Role of Job Satisfaction and Organizational Commitment

Although it is widely acknowledged that the causal link between job satisfaction and organizational commitment is still highly disputed, the psychological contract theory suggests that job satisfaction can strongly predict organizational commitment (Pandey, 2010). For instance, employees’ with high job satisfaction may develop a strong sense of loyalty and mutual commitment soon after understanding and accepting organizational goals and values (Bufquin et al., 2017; Chordiya et al., 2017; Kim et al., 2017). Thus, it seems more plausible that the organizational commitment of public employees can be influenced at least in part by the levels of their job satisfaction. That is, public employees with high levels of satisfaction with their jobs will more likely become committed to their work and organizations. According to psychological contract theory, when employees are satisfied with their pay, work autonomy, career training, and career development, they will become more committed to the organization, which in turn brings about positive performance behavior.

As mentioned above, a large body of research has confirmed that employees’ turnover intention can be significantly predicted by both job satisfaction and organizational commitment (Karatepe and Kilic, 2007; Kang et al., 2014; Mathieu et al., 2016; Wombacher and Felfe, 2017). For example, according to Vandenabeele (2009), job satisfaction and organizational commitment by state civil servants acted as mediators in the PSM–performance relationship. These results were further supported by Jin et al. (2018), who argued that P-O fit and organizational commitment mediated the link between PSM and organizational citizenship behavior. Furthermore, some empirical studies proposed structural turnover models that incorporated both job satisfaction and organizational commitment in a single model (Mathieu et al., 2016; Redondo et al., 2019). Therefore, based on the aforementioned discussions and hypotheses, we expect that job satisfaction and organizational commitment will play a chain-mediating role between PSM and turnover intention. Namely, PSM will help increase employees’ levels of job satisfaction first, which, in turn, has a positive effect on employees’ loyalty and organizational commitment, and then the positive work-related attitudes can effectively decrease employees’ intention to quit their jobs. Therefore, it leads to the following hypothesis.

*Hypothesis 6:* Job satisfaction and organizational commitment mediates the association between PSM and employees’ turnover intention sequentially.

## Materials and Methods

### Samples and Procedure

Data for this study were collected from MPA (master of public administration) students of two prestigious universities in Yunnan province, China, from August to September in 2019. Before the survey, we contacted the director of MPA Education Center of each university to obtain the total number of MPA students. With the help of staff of MPA Education Centers, we successively distributed survey questionnaires to 600 MPA students who are full-time public employees in various government departments. Each participant was asked to fill out the self-reported questionnaire voluntarily and anonymously. After deleting 13 questionnaires with missing data, we obtained a diverse sample of 587 participants who were full-time civil servants employed by local governments at all levels across Yunnan province. Of the participants, 306 (52.1%) were men and 281 (47.9%) were women; 42.2% of the participants were 30 years old or under and 13.1% were over 50 years old. For educational background, 74.8% held at least a bachelor’s degree, while 25.2% had an associate degree or less. In terms of length of employment, 31.2% worked for under 5 years, 23.5% worked for 6–10 years, 8.7% worked for 11–15 years, 7% worked for 16–20 years, and 29.6% worked for more than 21 years.

### Measures

#### Public Service Motivation

Public service motivation was measured with a translated version of five items developed by Perry (1996), which was widely used in various cultural contexts (Christensen and Wright, 2011; Qi and Wang, 2018). This 5-item scale was used to capture four aspects of individual PSM which included self-sacrifice (two items), commitment to public interests (one item), compassion (one item), and social justice (one item). A sample item was “Making a difference in society means more to me than personal achievements.” Each item is measured on a 5-point Likert scale that ranged from 1 (*strongly disagree*) to 5 (*strongly agree*). The Cronbach’s alpha was 0.835.

#### Job Satisfaction

Job satisfaction was evaluated with Boateng and Hsieh (2019) scale, including 4 items. Sample items included such statements as “No matter what, I will not leave my current job” and “Overall, I am satisfied with my current job.” Participants answered items on a 5-point Likert scale that ranged from 1 (*strongly disagree*) to 5 (*strongly agree*). The Cronbach’s alpha was 0.839.

#### Organizational Commitment

Organizational commitment was measured by Meyer et al. (1993) scale. We adopted four items to evaluate public employees’ organizational commitment. Sample items included “I feel emotionally attached to my organization.” All items are rated on a 5-point Likert scale that ranged from 1 (*strongly disagree*) to 5 (*strongly agree*). The Cronbach’s alpha was 0.884.

#### Turnover Intention

Turnover intention was assessed using the 4-item scale validated by Kim et al. (1996). Sample items included “I intend to leave my organization” and “I intend to stay in my present organization as long as possible.” Each item is rated on a 5-point Likert scale that ranged from 1 (*strongly disagree*) to 5 (*strongly agree*). The Cronbach’s alpha was 0.858.

### Analytical Strategy

We conducted data analyses using SPSS and AMOS version 24. The missing values were handled by using the full information maximum likelihood method before our analysis. According to the recommendation of Anderson and Gerbing (1988), a 2-step analytical strategy was used to conduct data analysis. First, to determine whether all study variables were distinguishable in the present study, we tested and compared the model fit of four models using confirmatory factory analysis (CFA). We assessed the hypothesized model compared with alternative models through confirmatory factory analysis. In addition, to avoid a risk of common method bias from as self-administered survey, the common method bias was tested by using Harman’s single-factor analysis.

Second, to test the hypotheses, structural equation modeling in AMOS 24 was performed to verify whether the relationship between PSM and turnover intention was mediated by job satisfaction or organizational commitment. Before testing the mediating effects, we first tested the direct effect of PSM on employee’ turnover intention without inclusion of the mediators in a structural equation model. Then, we used the biased-corrected bootstrapping method of the mediation test to analyze the indirect effects and mediated effects. As suggested by Preacher and Hayes (2008), the mediating effects are statistically significant when zero does not lie in the confidence interval range.

## Results

### Descriptive Analysis

The descriptive, correlations, and reliability for all study variables were shown in Table 1. According to the correlation matrix, PSM was negatively correlated with turnover intention (*r* = −0.370, *p* < 0.01), thus Hypothesis 1 was supported. In addition, PSM was positively related to job satisfaction (*r* = 0.437, *p* < 0.01) and also to organizational commitment (*r* = 0.418, *p* < 0.01), thus supporting Hypotheses 2 and 5. Both organizational commitment (*r* = −0.701, *p* < 0.01) and job satisfaction (*r* = −0.662, *p* < 0.01) were all negatively related to turnover intention, which supported Hypotheses 6 and 3. In addition, the construct coefficient alphas were above the minimum threshold value of 0.70, which ranged from 0.835 to 0.858, and suggested high reliability of the study scale.

**TABLE 1:** Means, standard deviations, correlations, and Cronbach’s Alpha Values among the study variables.

Variables	*1*	*2*	*3*	*4*	*5*	*6*	*7*	*8*
1. Gender	1							
2. Age	0.088*	1						
3. Education	0.044	0.073	1					
4. Tenure	0.108**	0.688**	0.289**	1				
5. PSM	0.079	0.029	0.012	–0.039	(0.835)			
6. JS	–0.064	−0.125**	–0.068	−0.282**	0.437**	(0.839)		
7. OC	–0.069	−0.117**	–0.077	−0.286**	0.418**	0.698**	(0.884)	
8. TI	0.119**	0.170**	0.050	0.334**	−0.370**	−0.662**	−0.701**	(0.858)
Mean	0.521	2.847	3.106	2.804	3.703	3.469	3.592	2.477
SD	0.5	1.081	0.759	1.644	0.633	0.726	0.776	0.764

*N* = 587, *p < 0.05, **p < 0.01. SD, standard deviation; PSM, public service motivation; JS, job satisfaction; OC, organizational commitment; TI, turnover intention. Figures in the bracket represent Cronbach’s Alpha Values.

### Confirmatory Factor Analysis

The goodness fit of the four-factor model was examined using confirmatory factor analysis (CFA). The model fit was assessed using three fit indices, including CFI (comparative fit index), TLI (Tucker-Lewis index), and RMSEA (root mean square error of approximation). We proposed five alternative model conceptualizations (Table 2). Compared with the five alternative models, the four-factor model resulted in a more acceptable fit [χ^2^ (113) = 281.432, *p* < 0.001; CFI = 0.970, TLI = 0.963, and RMSEA = 0.05]. For example, the fit indices in the two-factor model demonstrated that the model did not provide an adequate fit [χ^2^ (118) = 1433.392, *p* < 0.001; CFI = 0.753, TLI = 0.727, and RMSEA = 0.138] as well as the hypothesized four-factor model. The one-factor model provided a less adequate fit [χ^2^ (119) = 1580.686, *p* < 0.001; CFI = 0.736, TLI = 0.699, and RMSEA = 0.145]. Thus, the hypothesized four-factor model exhibited excellent construct distinctiveness in the present study.

**TABLE 2:** Comparison of measurement models for variables studied.

Model	*χ^2^*	*df*	*CFI*	*TLI*	*RMSEA*	*Δχ^2^(Δdf)*
Hypothesized four-factor model:	281.432	113	0.970	0.963	0.050	
**Three-factor model**						
Combining PSM with JS	1069.962	116	0.828	0.798	0.118	788.53(3)
Combining PSM with OC	1133.752	116	0.817	0.785	0.122	852.32(3)
Combining PSM with TI	1224.628	116	0.800	0.766	0.128	943.196(3)
Two-factor model (combining PSM, JS, and OC)	1433.392	118	0.753	0.727	0.138	1151.96(5)
One-factor model (combining all constructs)	1580.686	119	0.736	0.699	0.145	1299.254(6)

**N* = 587. TLI, Tucker-Lewis index; CFI, comparative fit index; RMSEA, root mean square error of approximation.*

### Testing of Common Method Bias (CMB)

Because the self-reported data was mainly collected from a single source, there was a risk of common method bias, such as social desirability and consistency motif (Podaskoff et al., 2003). To minimize the potential for common method bias, we adopted measures from previous studies and conducted a pre-test. Moreover, all respondents were informed to finish the questionnaire anonymously, which reduced the possibility of social desirability. To examine whether common method variance (CMV) bias was a concern, we assess the extent of common method bias in the present study by using a Harman’s single-factor test. The result indicated that 33.98% of the variance was explained by the first factor, which suggested the common method variance bias was not a problem in the present study. Furthermore, our results indicated a very poor fit in the one-factor model [χ^2^ (119) = 1580.686, *p* < 0.001; CFI = 0.736, TLI = 0.699, and RMSEA = 0.145]. Therefore, it also indicated that the common method bias was an unlikely interpretation of our findings.

### Hypothesis Testing

To test the study hypotheses, the fit of the data was tested using structural equation modeling (SEM). First, we tested the direct effect of PSM on turnover intention without inclusion of the mediators in the path structural equation model. PSM had a direct, negative effect on turnover intention (PSM→TI; *p* < 0.001, standardized estimate = 0.407), which supported hypothesis 1. Also, 54% of the variance in turnover intention (*R*^2^ = 0.54) was explained by PSM. This finding was consistent with the previous studies of samples from street-level bureaucrats in South Korea (Shim et al., 2017) and high school employees in the United States (Choi and Chun, 2018).

**FIGURE 1:**
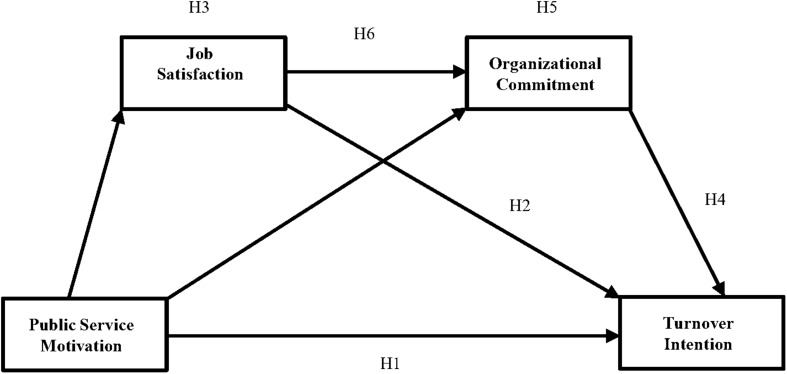
Research framework and hypotheses.

**FIGURE 2:**
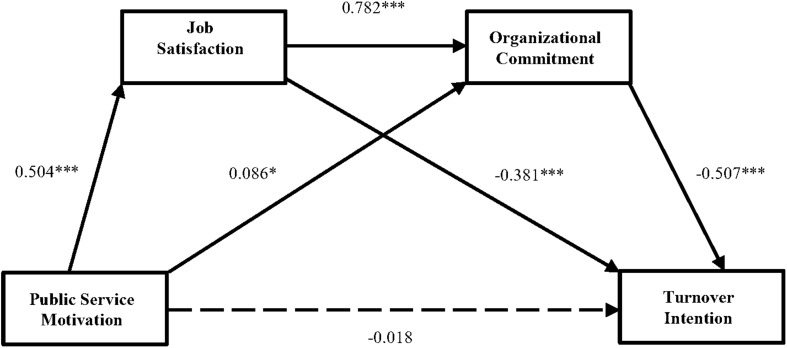
The mediation analysis of job attitudes between PSM and turnover intention (dotted lines represent relationships which are not significant in the full model), *p < 0.05, ***p < 0.001.

Then, we constructed a proposed model (Figure 1), which included the mediators. The results of the hypothesized model were shown in Table 3 and Figure 2 which provided standardized parameters and R^2^ coefficients. The results in Figure 2 showed that the fit indices for the hypothesized model were excellent [χ^2^ (111) = 246.104, *p* < 0.001; CFI = 0.976, TLI = 0.970, and RMSEA = 0.046], because CFI and TLI all are above 0.9, and RMSEA was at or below 0.06. As displayed, PSM had a direct, positive effect on job satisfaction (PSM→JS; *p* < 0.001, standardized estimate = 0.504) and organizational commitment (PSM→JS; *p* < 0.05, standardized estimate = 0.086). Furthermore, the direct effects of job satisfaction (JS→TI; *p* < 0.001, standardized estimate = −0.381) and organizational commitment (OC→TI; *p* < 0.001, standardized estimate = −0.507) on turnover intention were all negative and significant, which supported Hypotheses 2 and 4. In addition, job satisfaction positively affected organizational commitment (JS→OC; *p* < 0.001, standardized estimate = 0.782). However, PSM had no direct effect on employees’ turnover intention (standardized estimate = −0.018, *p* > 0.05) when the mediators were included in the hypothesized model.

**TABLE 3:** Direct and indirect effects from structural model.

	Direct effect			Indirect effect		
	→ JS	→ OC	→ TI	Estimate	95% Confidence interval	
					Lower	Upper
PSM	0.504***	0.086*	–0.018			
	(0.042)	(0.050)	(0.040)			
JS		0.782***	−0.381***			
		(0.038)	(0.093)			
OC			−0.507***			
			(0.092)			
PSM → JS → TI				−0.192	−0.970	−0.314
PSM → OC → TI				−0.043	−0.303	−0.010
JS → OC → TI				−0.397	−0.552	−0.242
PSM → JS → OC → TI				−0.120	−1.119	−0.442
R^2^	0.25	0.69	0.70			

*N* = 587, *p < 0.05, ***p < 0.001. PSM, public service motivation, JS, job satisfaction, OC, organizational commitment, TI, turnover intention.

To detect the indirect effects, a bootstrapping method was performed to test the indirect effects and mediating effects in the path structural equation model. As can be seen from Table 3 and Figure 2, the indirect impact of PSM on turnover intention through job satisfaction (PS→JS→TI) was statistically significant (standardized estimate = −0.192, CI = −0.970, −0.314), since the confidence interval range did not contain zero. Thus, job satisfaction fully mediated the negative relationship between PSM and turnover intention, which supported Hypothesis 3. Likewise, the indirect effect of PSM on turnover intention through organizational commitment (PSM→OC→TI) was statistically significant (standardized estimate = −0.043, CI = −0.303, −0.010), which indicated the mediating effect of organizational commitment in the association between PSM and employees’ intention to quit their jobs. Accordingly, Hypothesis 5 was supported. The bootstrapping test also presented an interesting finding that the indirect impact of job satisfaction on turnover intention through organizational commitment (JS→OC→TI) was significant (standardized estimate = −0.397, CI = −0.552, −0.242). This result indicated that the association between employees’ job satisfaction and their turnover intention was partially mediated by organizational commitment.

Finally, we tested whether employees’ job satisfaction and organizational commitment will sequentially mediate the path between PSM and turnover intention. As expected, the indirect effects of PSM on turnover intention through job satisfaction and organizational commitment (PSM→JS→OC→TI) was also statistically significant (standardized estimate = −0.120, CI = −1.119, −0.442), which confirmed that PSM negatively affected turnover intention through job satisfaction and organizational commitment in serial. Hence, Hypothesis 6 was supported.

## Discussion

In the present study, we examined how PSM influenced employees’ turnover intention in the public sector. The present study aimed to analyze the associations among PSM and the work-related variables that included job satisfaction, organizational commitment, and turnover intention. We also tested how employees’ job attitudes mediated the link between PSM and employees’ turnover intention.

First, employees’ with high PSM levels were less likely to leave their job within the public sector. In fact, the high-PSM employees were more likely to work in the public sector. A possible reason why public employees with high PSM levels can markedly lower the levels of turnover intention is because public employees with high PSM levels prefer to do these jobs that help others, serve the community of people and correct social inequity. Previous research has shown that public employee with high PSM levels place more importance on their jobs and showed more pro-social behaviors (Vandenabeele, 2008; Choi and Chun, 2018). Our results supported previous findings that public employees’ PSM had a direct, negative effect on their turnover intention (Perry and Wise, 1990; Shim et al., 2017).

However, the results suggested that employees’ PSM could not directly affect their turnover intention when job satisfaction and organizational commitment were taken into account. Consequently, our results supported a similar finding proposed by Bright (2008) that PSM had no direct, significant effect on employees’ turnover intention after mediators (e.g., P-O fit) were considered. Furthermore, our results were also similar to those suggested by Vandenabeele (2009), where job satisfaction, normative organizational commitment, and affective organizational commitment played a partial or full mediating role in the links between PSM and individual performance. Although some scholars stressed the significant direct effects of PSM on job attitudes, such as job satisfaction, and work-related behaviors (Kim, 2012; Jin et al., 2018), the indirect effect of PSM on turnover intention remains mixed when mediators are taken into account. The inconsistent association between PSM and turnover intention may depend on cultural context, which might be related to individuals’ PSM. More specifically, employees in collectivistic countries (e.g., China and Japan) would be more inclined to over-report their levels of PSM (Kim and Kim, 2016), because they are likely to hide their true intention to leave their job due to the profound influence of Confucianism.

Second, our study confirmed that the association between PSM and turnover intention was fully mediated by both job satisfaction and organizational commitment. Wright and Pandey (2008) demonstrated that the direct influence of PSM on work-related attitudes was mixed, so mediating variables were needed to explain this. As the results revealed, the effect of employees’ PSM on turnover intention was fully mediated by job satisfaction. In other words, employees with high PSM level experienced high level of job satisfaction, which led them to lower their intention to leave the public sector. Such findings are consistent with some previous results, which found that the positive PSM-job satisfaction association decreased employees’ intention to leave the public sector (Choi and Chun, 2018; Kjeldsen and Hansen, 2018). Therefore, jobs in the public sector may bring more satisfaction and ultimately lower their turnover intention for those employees with high levels of PSM. Likewise, organizational commitment played a fully mediating role between employees’ PSM and their turnover intention. Previous research revealed that “PSM had a direct, positive effect on organizational commitment” (Kim, 2012; Austen and Zacny, 2015; Caillier, 2016), thus, employees with high PSM levels showed strong commitment and loyalty to their organizations.

Third, one interesting finding was that organizational commitment partially mediated the negative association between employees’ job satisfaction and their turnover intention. Our result showed that job satisfaction indirectly affected employees’ turnover intention through organizational commitment. Actually, some previous studies have shown that job satisfaction significantly predicts organizational commitment (Bufquin et al., 2017); thus, we concluded that job satisfaction showed an indirect impact on turnover intention through its influence on organizational commitment. Our results suggested that employees with high levels of job satisfaction showed greater commitment than others to their public organizations, and they chose not to leave their current job within the public sector.

Lastly, our results showed that job satisfaction and organizational commitment played multiple mediating effects in a negative association between PSM and employees’ intention to quit. In prior studies, work-related attitudes and behaviors are regarded as independent intervening variables in the link between PSM and job performance (Kim, 2012; Jin et al., 2018), but few studies have incorporated job satisfaction and organizational commitment into a single turnover model. Therefore, our study provided more comprehensive analysis of the process of the direct and indirect effects of PSM on public employees’ turnover intention, and it offered further evidence for turnover model including job satisfaction and organizational commitment, which supported the findings of Vandenabeele (2008). More specifically, individuals with high PSM level tend to be more satisfied with their job, work environment, and organizations. Then, this type of job satisfaction gradually increases employees’ commitment to their organizations, thereby lowering the intention to change jobs within the public sector.

### Theoretical Implications

Our study contributes to the previous literature concerning the mixed link between PSM and turnover intention. First, our study contributes to understanding the disputed association between PSM and turnover intention by extending the existing PSM literature in the Chinese context. Although previous studies have revealed an inconsistent relationship between PSM and turnover intention (Bright, 2008; Kim, 2012; Shim et al., 2017), there is no denying that individual levels of PSM differ across nations and cultures that are viewed as an antecedent of PSM (Ritz and Brewer, 2013; Anderfuhren-Biget et al., 2014; Kim, 2017). Most previous studies verified the causal effect of PSM on turnover intention with samples from Western countries, but few studies were conducted in Asian countries with a Confucian culture, especially in China where such studies are virtually non-existent. Indeed, recent findings have confirmed the significant effects of national culture on individual levels of PSM in collectivistic countries (Ritz and Brewer, 2013; Kim, 2017). As a result of the influence of Confucian culture, Chinese public employees may tend to over-evaluate their levels of PSM, but they may hide their true intention to quit a job to the extent possible. As such, our study not only confirmed whether the effect of PSM on turnover intention was significant in a Chinese context, but it also tested the generalizability of Perry’s PSM theory. Given that cultural and institutional context play an important role in shaping individual levels of PSM, the reason for the complex links between PSM and turnover intention or other work-related behaviors might be interpreted from a cross-cultural perspective. Consequently, in addition to examining the association between PSM and individual work-related behavior, scholars might explore further how the overall relationship found in our study varies across cultural and institutional contexts. In a word, our study broadens previous research on the mixed association between PSM and turnover intention in a cultural context. The fact that our study took into consideration the cultural context in explaining the effects of PSM on work-related behaviors also offers a more comprehensive insight for understanding PSM theory.

Second, our study integrated job satisfaction and organizational commitment into a single turnover model, which resulted in a better understanding of the relation between PSM and turnover intention. To our knowledge, only three empirical studies in the literature have proposed a turnover model that incorporated both job satisfaction and organizational commitment (Williams and Hazer, 1986; Mathieu et al., 2016; Redondo et al., 2019). As mentioned above, most scholars have explained the causality between PSM and work-related performance using P-O fit theory (Gould-Williams et al., 2015; Jin et al., 2018), but few studies have attempted to include both job satisfaction and organizational commitment in a single turnover model. Perry et al. (2010) pointed out that scholars should pay careful attention to the roles of intervening variables with regard to the complicated PSM-performance relationships (Perry et al., 2010). In addition, some scholars have pointed out that PSM acts as a generic antecedent of job satisfaction and organizational commitment (Judge et al., 2001). Thus, we argued in this study, however, that the mediating role of public employees’ job attitudes in a turnover model should not be ignored. By doing so, our study theoretically broadens the mechanism through which PSM affects public employees’ turnover intention and might shed more light on the theoretical approaches to the disputed link between PSM and work-related behaviors.

### Practical Implications

Beyond the theoretical contribution to PSM theory, the present study offers significant insights for public organizations. First, since job applicants with high-PSM have a preference for the jobs within the public sector and are less likely to quit their job (Carpenter et al., 2012; Choi and Chun, 2018), our study indicates that job applicants with high PSM levels should be valued during the recruitment and selection procedures. To be more specific, the PSM index may be “used as one of the criteria in the employee selection process” (Lee and Choi, 2016) because it is urgent for public organizations to attract more individuals with the spirit of public concern and community values.

More importantly, the policy makers in the public organizations should pay close attention to the cultivation and promotion of employees’ levels of PSM through formal training and occupational socialization. Lee and Choi (2016) argued that PSM could be cultivated and increased through a variety of devices, therefore, the policy makers in the public organizations could adopt some effective ways to imbue public employees with underlying organizational values (Kim, 2012). In particular, in view of various cultural backgrounds of new public employees, a kind of effective formal training might cause them to appreciate organizational values and expectations that embody public interests and public service values (Paarlberg et al., 2008).

Furthermore, our findings also suggest that job satisfaction significantly predicts public employees’ turnover intention and, thus, it might provide some reasons for policy makers in public organizations to increase public employees’ salary and improve their treatment on the job. Actually, our results showed that job satisfaction was positively associated with employees’ organizational commitment and, in turn, led to employees’ intention to stay in the public sector. Therefore, one important revelation is that a kind of diversified incentive mechanism should be established in the public organization to enhance public employee’s job satisfaction. These incentive mechanisms could include not only material excitation means, but some non-material motivating measures, such as job promotion, career development, skills training, and job autonomy (Ni and Wu, 2013; Tang et al., 2017).

### Limitations and Future Research

As with any research, our study also has some limitations that should be taken into account in the future research. First, our study relied on a cross-sectional design that cannot allow us to make causal statements with certainty. Some recent studies have explored how employees’ turnover intention changes in accordance with affective commitment and job satisfaction based on longitudinal data (Chen et al., 2011; Vandenberghe et al., 2011). As such, future research should be based on longitudinal data that can further validate the causality between PSM and turnover intention. In particular, future studies should focus on how the trajectories of individual changes in PSM affect employees’ turnover intention and other work-related behaviors.

Second, we adopted a shortened version of Perry’s PSM scale, so the results of our study should be interpreted with caution. Although previous studies have widely verified the reliability of a shortened version of Perry’s PSM scale (Pandey et al., 2008; Wright and Pandey, 2008; Coursey et al., 2012), it would be worthwhile to develop an original PSM measure based on the Chinese context in future studies. As Liu (2009) argued, one of the PSM subdimensions—compassion dimension—could not be supported in the Chinese context. Future studies, therefore, are needed to propose and to validate a PSM scale consistent with Chinese cultural and institutional contexts.

Third, the data in the present study were collected from a single source, which means there was a risk of common method variance. In particular, it was possible that public employees answered questions in a similar way due to the influence of “official-oriented” thought and social desirability. In addition, because our data were derived from a sample collected from public employees in a single Chinese area, the sample of this survey does not completely match the characteristics of public employees’ nationwide. Therefore, although the common method bias is not a concern in our study, it needed to collected data from multiple sources in order to reduce potential common method bias.

Finally, given that PSM might change across cultures (Kim, 2017; Lee et al., 2020), it must be cautious to generalize the findings from one region (e.g., Yunnan province) to other regions of China. It is quite probable that individuals’ PSM levels might vary with region because each region of China has diversified ethnic culture. It may be worthwhile to explore whether our finding can be generalized in other regions of China with different ethnic culture and institution context. Thus, future research should verify the generalization of our findings with empirical data collected from different regions.

## Conclusion

Our study aimed to explore the mediating role of job satisfaction and organizational commitment to explain the association between public employees’ PSM and their turnover intention. Although public management scholars have confirmed the critical role of PSM in shaping individual work-related behavior (Shim et al., 2017; Choi and Chun, 2018; Jin et al., 2018), the results remains mixed regarding the relationship between PSM and turnover intention. This study provides additional insight into the link between PSM and turnover intention by proposing a chain moderating role of job satisfaction and organizational commitment. Our findings revealed that public employees’ PSM had no direct effect on their turnover intention when job satisfaction and organizational commitment were considered simultaneously. In addition, the present study stressed the importance of exploring the mediating role of job satisfaction and organizational commitment to predict the effects of PSM on public employees’ turnover intention. Our findings also indicated that organizational commitment played a mediating role in the association between public employees’ job satisfaction and their turnover intention. Furthermore, PSM indirectly affected employees’ turnover intention through its influence on job satisfaction and organizational commitment in the causal chain.

Although our findings are not completely consistent with some previous studies (Vandenabeele, 2009; Kim, 2012; Jin et al., 2018), it is important to recognize that the causality of the PSM-turnover intention link might depend on various mediators and different cultural and institutional contexts. Actually, without considering the influence of particular cultural and institutional backgrounds, it is impossible to understand the inconsistent results of the causal link between PSM and turnover intention. In particular, PSM and job satisfaction within the public sector should be regarded as significant psychological resources for the enhancement of employees’ organizational commitment and reduction of their turnover intention. As such, we expect that this type of research will not only help test the generalization of PSM theory by undertaking cross-cultural studies, but it will underscore the need for public sector organizations to attach importance to public employees’ PSM and job satisfaction.

## Data Availability Statement

All datasets generated for this study are included in the article/Supplementary Material.

## Ethics Statement

This study was conducted with oral informed consent from all participants. All participants were informed to complete the survey confidentially and anonymously. Based on this assumption, ethical review and approval was not required for this kind of study in China.

## Author Contributions

YL and QW collected the data and performed the data analyses. K-PG drafted and revised the manuscript. All authors contributed to the final version of manuscript and approved it for publication.

## Conflict of Interest

The authors declare that the research was conducted in the absence of any commercial or financial relationships that could be construed as a potential conflict of interest.
