# Supplementary material for: Public Service Motivation and Turnover Intention: Testing the Mediating Effects of Job Attitudes
Source: Front Psychol. 2020 Jun 23;11:1289. doi: 10.3389/fpsyg.2020.01289 (PMC7325603; doi:10.3389/fpsyg.2020.01289)
Supplement: Supplementary file 1 [file Data_Sheet_1.PDF]

Table 1. Description for each item of all measures (N = 587).

Items	Minimum	Maximum	Mean	SD
**Job satisfaction (Boateng and Hsieh, 2019)**				
overall, I am satisfied with my current job	1.00	5.00	3.3867	.87944
I have plans to look for a better job elsewhere (reverse coded)	1.00	5.00	3.6746	.82913
no matter what, I will not leave my current job	1.00	5.00	3.2726	.92591
I hate my current job (reverse coded)	1.00	5.00	3.5400	.90109
**PSM (Perry, 1996)**				
B2 Meaningful public service is very important to me.	1.00	5.00	3.8620	.67856
B6 I am often reminded by daily events about how dependent we are on one another.	1.00	5.00	3.8722	.68556
B12 Making a difference in society means more to me than personal achievements.	1.00	5.00	4.1516	.60361
B13 I am prepared to make enormous sacrifices for the good of society.	1.00	5.00	4.0239	.63416
B14 I am not afraid to go to bat for the rights of others even if it means I will be ridiculed.	2.00	5.00	4.0664	.58188
**Organizational commitment (Meyer et al,.1993)**				
I feel a strong sense of belonging to my organization	1.00	5.00	3.5400	.87222
my organization has a great deal of personal meaning for me	1.00	5.00	3.5673	.87054
I feel emotionally attached to my organization	1.00	5.00	3.9813	.66711
I feel like part of the family in my organization.	1.00	5.00	3.6678	.84278
**Turnover intention (Kim et al., 1996)**				
I intend to leave my organization	1.00	5.00	2.3867	.96103
I intend to stay in my present organization as long as possible	1.00	5.00	2.5315	.92939
In general, I will not take the initiative to leave the current unit	1.00	5.00	2.2811	.82889
If I stay in this unit, my future may not be better	1.00	5.00	2.7104	.92626

Table 2. Descriptive statistics of participants in the present study (N = 587).

Variables	Categories	N	%	Variables	Categories	N	%
Gender	Male	306	52.1	Child	With children	389	66.3
	Female	281	47.9		without children	198	33.7
Age	18-25	40	6.8	Work tenure	5 years or less	183	31.2
	26-30	208	35.4		6-10 years	138	23.5
	31-40	234	39.9		11-15 years	51	8.7
	41-50	28	4.8		16-20 years	41	7.0
	51 or older	77	13.1		21years or more	174	29.6
Marital status	Single	364	62.0	Working hours	8 hours or less	164	27.9
	Married	207	35.3		8-12 hours	406	69.1
	Divorced/Widowed	16	2.7		12 hours or more	17	2.9
Education	High school or below	31	5.3	Managerial level	Non-management	355	60.5
	Diploma	117	19.9		Lower	151	25.7
	Bachelor	268	45.7		Middle	71	12.1
	Masters/Doctoral	171	29.1		Upper	10	1.7
